# Enhancing Skills, Mood, and Performance in Overweight Handball Players: Exploring Individual vs. Collective Verbal Encouragement Strategies

**DOI:** 10.3390/children11040432

**Published:** 2024-04-03

**Authors:** Faten Sahli, Mohamed Mansour Bouzouraa, Mahmoud Rebhi, Amir Romdhani, Hajer Sahli, Atef Salem, Khaled Trabelsi, Achraf Ammar, Makram Zghibi

**Affiliations:** 1 Research Unit, Sportive Performance and Physical Rehabilitation, High Institute of Sports and Physical Education of Kef, University of Jendouba, Kef 7100, Tunisia; sehli.feten@gmail.com (F.S.); bouzouraamansour@gmail.com (M.M.B.); mahmoudrebhi@issepsf.u-sfax.tn (M.R.); makram.zghibi@issepkef.u-jendouba.tn (M.Z.); 2 High Institute of Sport and Physical Education of Sfax, University of Sfax, Sfax 3000, Tunisia; asalem@uni-mainz.de (A.S.); trabelsikhaled@gmail.com (K.T.); 3 Research Laboratory: Education, Motricity, Sport and Health, EM2S, LR19JS01, University of Sfax, Sfax 3000, Tunisia; romdhaniamir5@gmail.com (A.R.); sahlihajer2005@yahoo.fr (H.S.); 4 High Institute of Sport and Physical Education of Ksar Said, Manouba University, Manouba 2010, Tunisia; 5 Department of Training and Movement Science, Institute of Sport Science, Johannes Gutenberg University Mainz, 55122 Mainz, Germany; 6 Research Laboratory: Molecular Bases of Human Pathology, LR19ES13, Faculty of Medicine, University of Sfax, Sfax 3000, Tunisia; 7 Research Unit: Physical Activity, Sport and Health, UR18JS01, National Observatory of Sport, Tunis 1003, Tunisia

**Keywords:** coaching strategies, overweight students, sports performance, self-efficacy, team dynamics

## Abstract

This comparative study investigates the effectiveness of two teaching methods, individual verbal encouragement and collective verbal encouragement, in enhancing the technical–tactical skills and mood state of obese students during handball matches. This study employs a randomized controlled design and involves 28 overweight students (50% females), age: 17.4 ± 2.08 years and BMI: 26.8 ± 1.5 for females and 27.3 ± 2.1 for males. Technical–tactical skills are assessed through performance metrics (individual evaluation proposal by Gréhaigne) such as Ball Played (BP), Conquered Ball (CB), Lost Ball (LB), Shoots/Goals, Conservation index, and defensive index, while mood states are evaluated using pre- and post-tests (BRUMS Scale). Results reveal that individual verbal encouragement significantly enhances technical–tactical skills and positively influences the mood state of overweight students compared to collective verbal encouragement. Boys in Session 1 with VEI displayed a significantly higher number of ball plays (mean difference = 0.94 standard deviations, *p* = 0.004) and conquered balls (mean difference = 0.78 standard deviations, *p* = 0.006) compared to VEC. They also had a lower number of Lost Balls (mean difference = −0.62 standard deviations, *p* = 0.018) and a higher shooting efficiency (Shoots/Goals ratio, mean difference = 0.67 standard deviations; *p* = 0.013). Similar trends were observed in Session 2, with VEI, again, demonstrating advantages. Girls exhibited analogous improvements with VEI in both sessions. Notably, these performance enhancements coincided with positive emotional changes, with VEI leading to a greater decrease in depression and fatigue scores for both boys and girls. The study highlights the importance of tailoring teaching methods to the specific needs of overweight students in the context of handball, emphasizing the effectiveness of individualized verbal encouragement for skill development and emotional well-being. These findings offer practical implications for educators and coaches involved in physical education, advocating for personalized approaches to optimize learning experiences for overweight students in sports settings.

## 1. Introduction

The influence of verbal encouragement on the performance of student athletes, especially those classified as overweight, has become a critically important research focus. Numerous studies have emphasized the examination of the effects of this type of verbal motivation on the physical activity and engagement of student athletes, including those who are overweight.

In this context, studies have examined the effects of verbal encouragement during soccer dribbling drills in physical education settings [1]. This research aims to explore how verbal encouragement influences not only the physical aspects of performance, but also the psychophysiological responses of young adolescents. The objective was to emphasize the role of verbal encouragement in physical activities. 

Gonçalves et al. [2] and Hammami et al. [3] both contribute to our understanding of the effects of verbal encouragement in football, highlighting its influence on the strategic and tactical dimensions, as well as its physiological responses during gameplay. These findings are noteworthy as they highlight the potential of verbal cues to enhance both cognitive and physical aspects of performance in a sport that demands a combination of skills and endurance. However, it is essential to recognize that the specific mechanisms through which verbal encouragement exerts its effects may vary across different types of sports and individual athlete characteristics, warranting further investigation.

Similarly, Irwin et al. [4] and Puce et al. [5] extend this exploration beyond football, examining the motivational and performance-enhancing effects of verbal encouragement across various sports and activity settings. By considering the role of both coaches and peers in providing verbal support, these studies shed light on the social dynamics that influence athletes’ engagement and self-efficacy during gameplay. However, the generalizability of these findings may be limited by factors such as cultural differences in coaching styles and athletes’ perceptions of peer feedback.

Selmi et al.’s [6] investigation into the perception of effort adds another dimension to our understanding of verbal encouragement, highlighting its potential to influence individuals’ subjective experiences of exertion during physical activities. This study explores the importance of considering psychological factors in addition to physiological markers when evaluating the efficacy of verbal encouragement strategies. Nonetheless, future research should aim to elucidate the underlying mechanisms through which verbal cues modulate perceptions of effort and fatigue, particularly among individuals with varying levels of physical fitness and motivation.

Additionally, the studies focusing on overweight individuals, including those by Aydi et al. [7] and El-Gohary [8] offer valuable insights into the potential of verbal encouragement to enhance performance and participation among marginalized populations. By addressing the unique challenges faced by overweight individuals in engaging in physical activities, these studies highlight the importance of creating supportive environments that foster motivation and self-efficacy. However, they lack the identification of optimal strategies for delivering verbal encouragement tailored to the specific needs and preferences of overweight individuals, taking into account factors such as body image concerns and past experiences with physical activity.

Furthermore, the studies by Hawani et al. [9] and McWhorter et al. [10] highlight the broader implications of verbal encouragement for promoting physical activity and improving psychophysiological outcomes among overweight students. These findings suggest that verbal cues may serve as a powerful tool for addressing the complex interplay of psychological and physiological factors that contribute to obesity-related health disparities.

Except for the study by Sahli et al. [11], which demonstrated the beneficial effects of learning with verbal encouragement, compared to learning with compliments from the teacher, no research has explored the modalities of encouragement while students are learning, specifically examining the difference between individual verbal encouragement for each overweight student and collective encouragement for the entire team, irrespective of individual players.

The present study aims to investigate the effectiveness of different verbal encouragement approaches on enhancing skills, mood, and performance among overweight handball players. Building upon existing research, this study seeks to address the unique challenges faced by overweight individuals participating in competitive sports, particularly handball, by focusing on tailored verbal encouragement strategies.

The first hypothesis posits that individual verbal encouragement provided by coaches or instructors could lead to improvements in specific skill performance metrics and enhance athletes’ mood states. This hypothesis is justified by previous research demonstrating the positive impact of personalized feedback and motivation on skill acquisition and psychological well-being among athletes. By providing tailored encouragement, coaches can create an environment conducive to optimal performance and positive mood states among overweight handball players.

In line with the first hypothesis, collective verbal encouragement, particularly from peers or teammates, is expected to result in improvements in overall team performance indicators and enhance athletes’ mood states. This hypothesis is supported by the understanding that peer support and camaraderie significantly impact team cohesion and performance outcomes in team sports. By fostering a supportive and cohesive team environment, collective verbal encouragement may play a crucial role in enhancing the overall sports experience for overweight handball players.

Through the empirical testing of these hypotheses, findings of the present study may inform the development of targeted interventions aimed at optimizing the sports experience and promoting overall well-being among individuals with obesity participating in competitive sports.

## 2. Materials and Methods

### 2.1. Subjects

A randomized and balanced design was implemented to ensure the unbiased and equal assignment of participants to various training groups.

An a priori power analysis was conducted using G*Power version 3.1.9.6 to determine the minimum sample size required to test the study hypothesis [12]. Results indicated the required sample size to achieve 80% power for detecting a small effect size, at a significance criterion of α = 0.05, was N = 22 for 2 × 2 repeated measures Analysis of Variance (ANOVA). Thus, the obtained sample size of 28 is adequate to test the study hypothesis. The research was conducted in the Kef region of Tunisia throughout January and February 2022, with participants consisting of (total = 28) 14 boys aged 17.5 ± 2.4 years, with a body mass of 89.7 ± 1.6 Kg, a height of 173.2 ± 1.5 cm, and an IMC of 27.3 ± 2.1; and 14 girls aged 17.3 ± 1.7 years, with a body mass of 88.2 ± 2.6 Kg, a height of 164.3 ± 2.7 cm, and an IMC of 26.8 ± 1.5; the students were affiliated with the Ahmed Amara school in the same region. Recruitment was voluntary, facilitated through a technical administration announcement, and selection was based on players’ availability within their schedules. To be eligible, participants had to meet specific criteria, including good health, absence of acute or chronic diseases, and no ongoing medical treatment. These criteria were crucial for ensuring participant safety and well-being. Prior to the study, all participants and their parents were fully informed about the potential risks and inconveniences associated with the experimental procedures. The informed consent process allowed parents to grant permission for their children’s participation, ensuring a comprehensive understanding of the study’s objectives and procedures by all involved parties.

### 2.2. Instruments

#### 2.2.1. Individual Evaluation Proposal by Gréhaigne

The use of the individual evaluation proposal by Laroche [13] involves recording behaviors deemed significant in adapting to a game model [14], whether for the team or individual players. For the latter, following the principle of “not losing the ball to bring it into the scoring zone and score”, a simple tally of the number of goals scored (B), the number of shot attempts (T), and the number of ball possessions (P) in matches of constant duration provides valuable insights. This allows the calculation of the number of balls won per unit of time in a situation of balanced power dynamics, potentially serving as a meaningful indicator of the level of play. Indeed, a higher ball exchange between the two teams suggests weaker teams with fewer goals or very skilled teams with numerous goals. To gather data on in-game occurrences and within the skill network, we analyzed different game sequences [15,16,17]. These sequences can be defined as the “ball exchanges” between players from gaining possession until its loss (goal or ball to the opponent) by the team. For example, in a team of four players, all ball exchanges within each team are documented, capturing every game event. This method allows for the precise identification of players making engagements and throw-ins, those losing or gaining possession of the ball, and those taking shots on goal. The volume of play signifies a player’s or team’s capacity to fully engage in a match in the quantitative sense of the term, reflecting their presence and activity in the competition. It is simply represented by the number of balls played (BP) within a specific power dynamic. Some definitions include balls played—any touched ball; balls conquered (BC)—intercepted or recovered balls through a foul (throw-in, free-kick, etc.); and lost-ball (LB)—balls leading to a game stoppage, a foul, or recovery by the opponent. The same player may have BP, BC, and LB for the same or a very similar action within a close timeframe.

#### 2.2.2. BRUMS Scale

The Arabic version of the modified Brunel Mood Scale (BRUMS), developed by the authors of [18], was utilized to assess the mood state of participants. This assessment involved participants responding to the question “How do you feel right now?” immediately after each session. The BRUMS is composed of six sub-scales, which include fatigue, anger, vigor, confusion, depression, and tension. These sub-scales consist of a total of 24 items, with each item being graded on a 5-point Likert-type scale. These items provide insights into various aspects of the emotional state of the student. For instance, to assess anger, respondents might consider statements like “I feel easily annoyed or frustrated today”. Confusion is evaluated through items such as “My mind feels muddled today”, while depression is measured using statements like “I feel downhearted and hopeless today”. Fatigue is captured by items like “I feel extremely tired today” and tension is assessed with statements such as “I feel tense and keyed up today”. On the other hand, vigor, or the presence of energy and vitality, is evaluated with items like “I feel full of energy today”. Together, these items offer a comprehensive overview of mood profile. The scale ranges from 0, indicating “not at all”, to 4, denoting “extremely” in terms of intensity. The raw score for each sub-scale is calculated by summing the responses of the four relevant items, resulting in a score range of 0 to 16 for each sub-scale.

The Arabic Brunel Mood Scale demonstrates satisfactory internal consistency reliability, meaning its different subscales measuring various mood aspects show consistent scoring. Studies evaluating its psychometric properties employed Cronbach’s alpha, a measure of internal consistency, with acceptable values exceeding 0.70. All six subscales, including anger, confusion, depression, fatigue, and tension, achieved alpha values above 0.70, indicating good consistency. Even vigor, the one subscale with a slightly lower value of 0.749, still fell within an acceptable range. These findings suggest the Arabic Mood Scale (ARAMS) is a reliable tool for assessing various mood aspects in Arabic-speaking populations, particularly those studied in physical education contexts [19].

### 2.3. Procedure

Before the initiation of the training sessions, anthropometric measurements were conducted, encompassing height, weight, body mass index (BMI), waist circumference, hip circumference, and waist-to-hip ratio for a cohort of 14 boys and 14 girls. There were two training sessions, consisting of two bouts each lasting twelve min, and each session was preceded by a warm-up involving low-intensity running, coordination movements, and dynamic stretching and ended with 4 × 8 m sprints; 3 min of recovery separated the warm-up from the first bout of the training session, which was designed to compare the impact of individual verbal encouragement (VEI) and collective verbal encouragement (VEC) during 7v7 handball matches. The VEI group received encouragement from the coach before each session and during recovery periods, whereas the VEC group received encouragement during the actual game. Technical–tactical skills, including layed ball, conquered ball, lost ball, shots/goals, conservation index, and defensive index, were systematically filmed using an HD SONY camera throughout each session. To gauge the participants’ mood states, the BRUMS scale was administered both before and after each session, including pre- and post-tests for each bout. This comprehensive approach integrates anthropometric measurements, training interventions, video analysis of technical–tactical skills, and mood state assessments, providing a holistic understanding of the impact of individual and collective verbal encouragement on handball performance and psychological well-being.

### 2.4. Data Analysis

The study’s data were presented in the form of means and standard deviations (M ± SD). The Shapiro–Wilk test was employed to assess the normality of the data distribution. A paired T-test was employed to compare the technical–tactical skills during the training sessions for overweight students between the individual and collective verbal encouragement groups. In contrast, a repeated measures ANOVA (2 × 2 time) was utilized to compare mood states during the pre- and post-tests for each session. The degree of change was categorized as trivial, small, medium, or large, based on Cohen’s d values ranging from 0 to 0.20, >0.20 to 0.50, >0.50 to 0.80, and >0.80, respectively. All statistical analyses were performed using the Statistical Package for the Social Sciences (SPSS, v28.0), with a significance level set at *p* < 0.05.

## 3. Results

### Technical–Tactical Skills

The provided data (Table 1) present a comprehensive analysis of various technical–tactical performance variables for boys in Session 1, comparing individual verbal encouragement (VEI) and collective verbal encouragement (VEC).

Overall, boys in Session 1 with VEI showed a significantly higher number of ball plays compared to VEC (*p* = 0.004; d = 0.94) and also had higher Conquered Ball values (*p* = 0.006; d = 0.78). Additionally, VEI had lower Lost Ball values (*p* = 0.018; d = −0.62) and a higher Shoots/Goals ratio (*p* = 0.013; d = 0.67). In Session 2, similar trends were observed, with VEI exhibiting a significantly higher number of ball plays (*p* = 0.005; d = 0.89) and Conquered Ball values (*p* = 0.009; d = 0.72), along with lower Lost Ball values (*p* = 0.004; d = −0.82) and a higher Shoots/Goals ratio (*p* = 0.001; d = 1.84).

For girls in Session 1, VEI displayed higher values in Balls played (*p* = 0.039; d = 0.50) and Conquered balls (*p* = 0.021; d = 0.59), while also reporting lower Lost Ball values (*p* = 0.045; d = −0.48) and a higher Shoots/Goals ratio (*p* = 0.02; d = 0.60). In Session 2, similar patterns were observed, with VEI showing significantly higher Balls played values (*p* = 0.016; d = 0.64) and Conquered balls values (*p* = 0.015; d = 0.65), as well as lower Lost Ball values (*p* = 0.03; d = −0.64) and a higher Shoots/Goals ratio (*p* = 0.002; d = 0.93) compared to VEC. 

**Figure 1:**
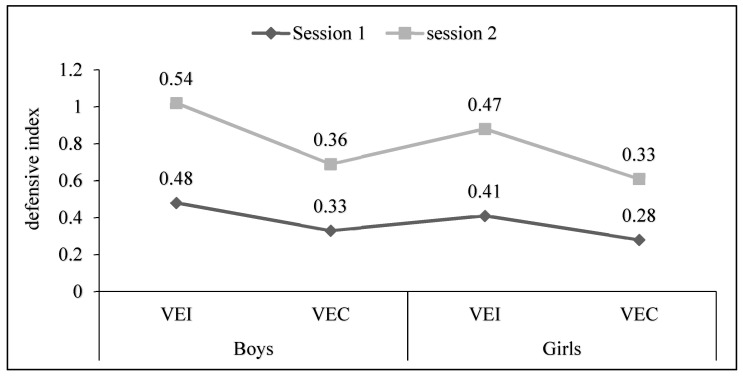
Score total of defensive index for each session between individual verbal encouragement and collective verbal encouragement (defensive index = Conquered Ball/Lost Ball).

Both in Session 1 and Session 2, the VEI group consistently exhibits a higher defensive index compared to the VEC group. Similar to the boys, girls in the VEI group consistently show a higher defensive index compared to the VEC group in both sessions. This indicates that girls receiving individual verbal encouragement tend to have a stronger defensive performance on average (Figure 1). Overall, the defensive index values suggest a potential positive impact of individual verbal encouragement on defensive performance, as reflected in the higher defensive index values, as compared to collective verbal encouragement across both boys and girls and in both sessions. Further context about the specific calculation of the defensive index and the scale used for interpretation would provide a more in-depth analysis.

**Figure 2:**
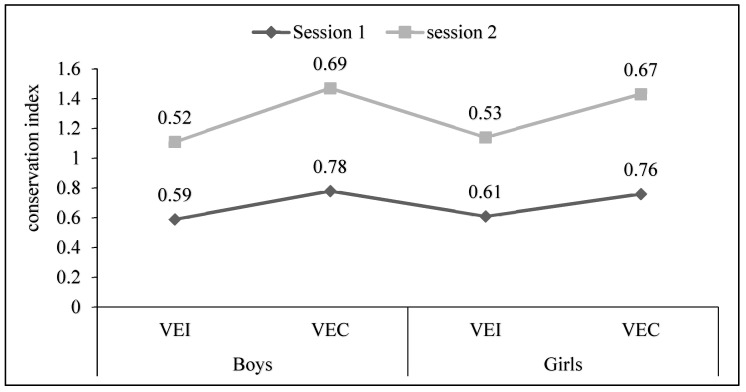
Score total of conservation index for each session between individual verbal encouragement and collective verbal encouragement (conservation index = Lost Ball/Played Ball).

In both Session 1 and Session 2, the VEI group consistently exhibits a lower conservation index compared to the VEC group. This suggests that, on average, boys in the VEI group show less conservation in the specified context. Similar to the boys, girls in the VEI group consistently have a lower conservation index compared to the VEC group in both sessions (Figure 2). This indicates that girls receiving individual verbal encouragement tend to exhibit less conservation on average. Overall, the conservation index values suggest a potential difference in conservation behavior between the VEI and VEC groups. However, the specific interpretation of these values would depend on the context and scale used for the conservation index.

An analysis was conducted to determine the internal consistency reliability of the Brunel Mood Scale (BRUMS) within our dataset. Our examination yielded notable Cronbach’s α coefficients for each dimension of the BRUMS, as follows: tension (α = 0.845), depression (α = 0.891), anger (α = 0.852), vigor (α = 0.912), fatigue (α = 0.839), and mental confusion (α = 0.905). These coefficients indicate strong internal consistency within each dimension, suggesting that the items within each subscale reliably measure their respective mood states.

The data from the repeated measures ANOVA, comparing the effects of VEI and VEC for boy players during a handball match on depression scores, reveal significant findings (Table 2). For both boys and girls, the repeated measures ANOVA revealed the significant effects of the intervention (VEI vs. VEC) on depression and fatigue scores. Boys who received VEI showed a greater decrease in depression and fatigue compared to VEC. Similarly, girls in the VEI group demonstrated a larger decrease in these scores. These findings, supported by significant interaction effects (*p* < 0.006), highlight the differential impact of verbal encouragement type on these emotional states.

For boys, VEI also led to a more significant decrease in confusion and tension scores, compared to VEC. The interaction effects for both emotions were significant (*p* < 0.05), suggesting the type of encouragement mattered. However, anger scores decreased similarly for both groups. Interestingly, boys in the VEI group exhibited a greater increase in vigor compared to VEC. Girls’ results mirrored the boys for depression and fatigue, with VEI showing a more substantial decrease. Additionally, VEI led to a larger decrease in confusion and anger scores compared to VEC, with significant interaction effects (*p* < 0.001). Similar to the boys, girls in the VEI group showed a greater increase in vigor compared to VEC (Table 3). These findings emphasize the importance of the specific type of verbal encouragement (VEI vs. VEC) in influencing emotional states and vigor in young athletes during handball matches.

## 4. Discussion

The study’s findings reveal a significant difference in the impact on technical–tactical skills between individual and collective verbal encouragement. Specifically, participants who received individualized verbal encouragement demonstrated a discernible improvement in their technical–tactical abilities compared to those in the collective verbal encouragement group. This divergence suggests that personalized feedback and motivation tailored to individual learners may be more effective in enhancing their performance [20]. The positive effect observed in the individual verbal encouragement condition could be attributed to the focused attention and specific guidance provided to each participant. In contrast, collective verbal encouragement, while fostering a sense of group unity, may lack the precision needed to address the diverse needs and skill levels within the cohort. This nuanced analysis underscores the importance of considering the individualized nature of motivational strategies in educational and coaching settings, emphasizing the potential superiority of tailored feedback over generalized encouragement for optimizing technical–tactical skill development [21].

These results highlight the efficacy of personalized and targeted motivational feedback in positively influencing the performance of overweight students in the realm of technical–tactical skills [10]. Individual verbal encouragement likely addresses the unique needs and challenges faced by each overweight student, providing specific guidance and motivation tailored to their capabilities [22]. In contrast, collective verbal encouragement, while fostering a sense of group unity, may not sufficiently address the individualized requirements of overweight students [23]. Individualized encouragement may directly address students’ specific emotional and psychological needs, thereby reducing feelings of tension and depression, while enhancing vigor, which is associated with energy and readiness to engage [24]. In contrast, collective verbal encouragement did not show the same level of positive impact on mood states. While it might foster a sense of belonging and group identity, it lacks the personalized touch necessary to fully meet individual psychological needs. This approach may inadvertently supervise the unique emotional challenges and barriers faced by overweight students, failing to provide the specific motivational cues and support needed to significantly alter mood states in a positive direction. Moreover, the study’s findings on mood states highlight the need for educators and coaches to adopt a better understanding of motivation and psychological support. 

The results indicated that individual verbal encouragement had a significantly more positive effect on the mood state of overweight students compared to collective verbal encouragement [11]. A psychological analysis suggests that the personalized and targeted nature of individual verbal encouragement likely resonates more effectively with the unique psychological needs of overweight students, fostering a sense of individualized support and motivation. This approach may enhance their emotional well-being by addressing specific challenges and providing tailored encouragement. In contrast, the collective verbal encouragement, while promoting a sense of group unity, might lack the specificity required to effectively address the diverse psychological responses within the cohort of overweight students [23]. These findings underscore the importance of considering individualized psychological strategies in educational and coaching environments to optimize the emotional well-being of overweight students.

It is important to consider individual differences when designing interventions to enhance performance. Piepiora and Naczyńska [25] investigated the relationship between personality traits and sports classes in junior sports acrobatics. Their findings suggest that athletes with different personality profiles might respond differently to training approaches. Similarly, our study suggests that individual versus collective verbal encouragement strategies may differentially impact mood and performance depending on the players’ personalities.

The findings from previous studies underscore the substantial impact of verbal encouragement on technical skills [3]. This suggests that verbal cues and motivational language play a crucial role in influencing the development and execution of technical skills in the context of the study. The positive effect observed could be attributed to the psychological aspects of encouragement, such as increased confidence, focus, and motivation, which are known to contribute to improved skill acquisition. Verbal encouragement may create a supportive and positive learning environment, enhancing the athlete’s receptivity to instruction and their overall performance [11]. Additionally, this underscores the significance of the coach’s role in providing effective and targeted verbal feedback to athletes, emphasizing the need for motivational strategies tailored to individual needs. These findings contribute valuable insights to the understanding of the intricate relationship between verbal encouragement and technical skill development, with potential implications for coaching practices and athlete training programs.

Additionally, several studies highlight the significant influence of verbal encouragement on the mood state of students in physical education [1]. These findings suggest that positive and motivating verbal cues can play a crucial role in shaping the emotional experiences of students during physical education activities. Verbal encouragement, when effectively employed by educators or coaches, has the potential to enhance students’ mood states by fostering a positive and supportive atmosphere [26]. This positive impact on mood may contribute to increased motivation, engagement, and enjoyment of physical activities, ultimately promoting a more favorable learning environment. The implications of these studies underscore the importance of incorporating intentional and encouraging communication strategies within the context of physical education to positively affect students’ emotional well-being.

Given the critical nature of the interaction between teachers and students [26,27,28], or between coaches and athletes [29,30], effective and targeted verbal encouragement is a powerful motivational instrument that can enable students and athletes to push their limits and achieve new heights of success. 

Educators and coaches who stand out are those who know how to discern motivational needs. Such an ability to provide constructive verbal encouragement is not a one-size-fits-all approach; this requires a nuanced understanding of the personal aspirations and emotional states of each student or athlete. By adapting their verbal encouragement in their communication style, educators and coaches can greatly influence and strengthen the teacher–student or coach–athlete relationship [31]. This adaptive communication is vital not only to improve performance, but also to forge a bond based on trust, respect, and mutual understanding. In similar educational and sporting contexts [32], this relationship is essential to fostering resilience in students and athletes [33]. This type of support provided is invaluable when individuals face challenges, setbacks, or failures.

Additionally, by cultivating a supportive environment, educators and coaches play a crucial role in encouraging a positive attitude toward continuous improvement and lifelong learning. Such an environment not only promotes the development of technical skills, but also contributes to the emotional and psychological well-being of students and athletes [34], which is equally important for their overall growth and development.

### 4.1. Implications

The findings of this study have significant implications for both practice and understanding within the realm of sports psychology. The delineation between individual and collective verbal encouragement strategies offers practical insights for coaches and practitioners working with overweight handball players. Tailoring interventions based on the effectiveness of these strategies can potentially enhance skills, mood, and overall performance among this population. 

Moreover, by emphasizing the importance of mood states alongside skill performance, the study underscores the intricate interplay between psychological factors and athletic success, particularly in individuals facing unique challenges such as obesity. Implementing effective verbal encouragement strategies not only fosters physical skill development, but also positively influences mood states, ultimately contributing to athletes’ psychological well-being. 

Additionally, the exploration of collective verbal encouragement highlights the pivotal role of team dynamics in sports settings. Cultivating a supportive team environment through peer encouragement may enhance cohesion, communication, and solidarity among overweight handball players, thereby potentially improving team performance and satisfaction.

### 4.2. Limitations

Several limitations warrant consideration. Firstly, the generalizability of the study’s findings may be constrained due to its focus on overweight handball players. While the insights gained are invaluable within this specific context, variations in sport-specific demands, individual characteristics, and environmental factors across different sports or populations may limit the direct applicability of the results. 

Secondly, since our sample size is small, it may be considered a limitation. While efforts were made to mitigate this limitation through rigorous methodology and statistical analysis, the small sample size may affect the generalizability of the findings and the robustness of the conclusions drawn. Future research with larger and more diverse samples would help strengthen the external validity of the study’s results and provide a more comprehensive understanding of the phenomena under investigation.

Moreover, the reliance on self-report measures and observational data for assessing mood states and performance outcomes introduces potential biases and measurement errors. Incorporating objective measures or alternative assessment methods could enhance the validity and reliability of future studies. Furthermore, the study’s short-term focus may overlook the potential long-term effects of verbal encouragement interventions. Investigating the sustainability of improvements in skills, mood, and performance over time, as well as exploring the underlying mechanisms driving these effects, is crucial for informing the development of effective long-term interventions in sports psychology.

## 5. Conclusions

In conclusion, this study investigated the efficacy of two teaching methods, individual verbal encouragement and collective verbal encouragement, in improving technical–tactical skills and mood state among overweight students during handball matches. Our findings indicate that individual verbal encouragement yielded superior outcomes compared to collective verbal encouragement, enhancing both skill development and emotional well-being. 

The personalized nature of individual encouragement appears to align more effectively with the needs of overweight students, contributing to their improved performance and mood state during physical activities. These results emphasize the importance of tailoring teaching methods to the specific requirements of overweight students in the context of sports, particularly handball. The implications of these findings extend to educators, coaches, and practitioners in physical education, suggesting the value of adopting personalized approaches to optimize the learning and participation experiences of overweight students in sports settings.

## Figures and Tables

**Table 1:** Comparison of technical–tactical skills during the game between individual verbal encouragement group and collective verbal encouragement group.

		Variable	VEI	VEC	95% CI		d	t-Value	*p*-Value
			M ± SD	M ± SD	Lower	Upper			
**Boys**	Session 1	Ball played	22.70 ± 2.10	19.83 ± 2.47	1.11	4.63	0.94	3.53	0.004
		Conquered Ball	6.51 ± 1.11	5.23 ± 1.19	0.33	2.23	0.78	2.91	0.006
		Lost Ball	13.54 ± 1.88	15.57 ± 1.82	−3.91	−0.14	−0.62	−2.32	0.018
		Shoots/Goals	11.59 ± 1.97	9.60 ± 1.59	0.27	3.71	0.67	2.50	0.013
	Session 2	Ball played	23.38 ± 2.67	20.49 ± 2.70	1.02	4.75	0.89	3.35	0.005
		Conquered Ball	6.71 ± 1.52	5.18 ± 1.67	0.31	2.73	0.72	2.73	0.009
		Lost Ball	12.36 ± 1.52	14.24 ± 1.81	−3.19	−0.56	−0.82	−3.08	0.004
		Shoots/goals	11.65 ± 1.20	9.84 ± 1.24	1.24	2.37	1.84	6.92	0.001
**Girls**	Session 1	Ball played	16.80 ± 2.33	15.01 ± 2.41	−0.23	3.80	0.50	1.91	0.039
		Conquered Ball	4.30 ± 1.19	3.23 ± 0.93	0.04	2.09	0.59	2.24	0.021
		Lost Ball	10.35 ± 1.64	11.53 ± 1.46	−2.56	0.21	−0.48	−1.83	0.045
		Shoots/goals	8.22 ± 1.70	6.43 ± 2.34	0.08	3.49	0.60	2.27	0.020
	Session 2	Ball played	17.74 ± 2.52	15.61 ± 2.72	0.22	4.02	0.64	2.41	0.016
		Conquered Ball	4.47 ± 1.22	3.49 ± 0.80	0.10	1.83	0.65	2.43	0.015
		Lost Ball	9.50 ± 1.52	10.56 ± 1.34	−1.99	−0.12	−0.64	−2.44	0.030
		Shoots/goals	8.29 ± 1.46	6.36 ± 1.72	0.73	3.12	0.93	3.49	0.002

Note: M ± SD = mean and standard deviation; VEI = individual verbal encouragement; VEC = collective verbal encouragement; d = Cohen’s d, t-value = calculated Student’s *t*-test value; 95% CI = 95% confidence interval.

**Table 2:** Descriptive statistics, mean and standard deviation (pre- and post-test for individual verbal encouragement group and collective verbal encouragement group.

		VEI		VEC	
		M ± SD		M ± SD	
		Pre-Test	Post-Test	Pre-Test	Post-Test
**Boys**	Depression	6.35 ± 1.00	3.28 ± 1.54	6.22 ± 1.42	5.42 ± 1.50
	Fatigue	7.29 ± 0.89	3.71 ± 1.58	7.14 ± 1.09	5.92 ± 1.49
	Confusion	6.78 ± 1.62	3.64 ± 1.69	7.07 ± 1.14	6.07 ± 1.59
	Tension	6.64 ± 1.69	3.74 ± 1.72	7.21 ± 1.25	6.14 ± 1.70
	Anger	7.73 ± 1.13	4.42 ± 1.94	7.35 ± 1.33	6.21 ± 1.76
	Vigor	8.35 ± 1.15	10.64 ± 1.33	6.85 ± 1.40	7.64 ± 1.44
**Girls**	Depression	7.85 ± 1.02	4.28 ± 2.05	7.38 ± 6.33	6.33 ± 1.90
	Fatigue	7.57 ± 1.22	3.64 ± 1.94	7.28 ± 1.26	6.57 ± 2.10
	Confusion	7.78 ± 1.42	2.85 ± 1.83	7.50 ± 1.50	6.85 ± 2.38
	Tension	8.00 ± 1.51	4.42 ± 2.82	8.07 ± 1.97	7.28 ± 2.72
	Anger	8.50 ± 2.02	4.14 ± 2.10	8.42 ± 2.34	7.85 ± 3.05
	Vigor	8.92 ± 1.73	11.42 ± 1.28	7.57 ± 1.78	8.07 ± 1.67

Note: M ± SD = mean and ± standard deviation; VEI = individual verbal encouragement; VEC = collective verbal encouragement.

**Table 3:** ANOVA repeated measure (2 × 2 time) comparison of mood state between individual verbal encouragement group (VEI) and collective verbal encouragement group (VEC).

		Training Effect			Time Effect			Interaction Effect		
		F_(1,26)_	η^2^	*p*	F_(1,26)_	η^2^	*p*	F_(1,26)_	η^2^	*p*
**Boys**	Depression	6.04	0.18	0.021	34.15	0.56	0.001	11.99	0.31	0.002
	Fatigue	4.52	0.14	0.043	81.98	0.75	0.001	10.69	0.29	0.003
	Confusion	8.75	0.25	0.007	34.81	0.57	0.005	9.31	0.26	0.005
	Tension	10.21	0.28	0.004	26.99	0.50	0.001	5.82	0.18	0.023
	Anger	2.43	0.08	0.13	29.39	0.60	0.001	8.86	0.25	0.006
	Vigor	27.60	0.51	0.001	32.26	0.55	0.001	7.69	0.22	0.010
**Girls**	Depression	10.27	0.28	0.004	57.30	0.68	0.001	30.20	0.53	0.001
	Fatigue	10.51	0.18	0.023	51.76	0.66	0.001	24.81	0.48	0.001
	Confusion	13.68	0.32	0.003	54.24	0.67	0.001	32.09	0.55	0.001
	Tension	4.58	0.15	0.042	15.86	0.37	0.001	6.48	0.20	0.017
	Anger	5.80	0.18	0.023	22.94	0.46	0.001	13.54	0.34	0.001
	Vigor	23.73	0.47	0.001	15.30	0.37	0.001	6.80	0.20	0.015

Note: Eta-squared (η2) is a measure of effect size for use in ANOVA.

## Data Availability

Data are available from the first author upon reasonable request.
